# Idiopathic and immune‐related pulmonary fibrosis: diagnostic and therapeutic challenges

**DOI:** 10.1002/cti2.1086

**Published:** 2019-11-05

**Authors:** Andrew McLean‐Tooke, Irene Moore, Fiona Lake

**Affiliations:** ^1^ Department of Clinical Immunology Sir Charles Gairdner Hospital Perth WA Australia; ^2^ Department of Laboratory Immunology PathWest QEII Medical Centre Perth WA Australia; ^3^ Department of Respiratory Medicine Fiona Stanley Hospital Perth WA Australia; ^4^ Department of Respiratory Medicine Sir Charles Gairdner Hospital Perth WA Australia

**Keywords:** connective tissue disease, diagnosis, interstitial lung diseases (ILDs), pulmonary fibrosis, therapeutics

## Abstract

Interstitial lung disease (ILD) encompasses a large group of pulmonary conditions sharing common clinical, radiological and histopathological features as a consequence of fibrosis of the lung interstitium. The majority of ILDs are idiopathic in nature with possible genetic predisposition, but is also well recognised as a complication of connective tissue disease or with certain environmental, occupational or drug exposures. In recent years, a concerted international effort has been made to standardise the diagnostic criteria in ILD subtypes, formalise multidisciplinary pathways and standardise treatment recommendations. In this review, we discuss some of the current challenges around ILD diagnostics, the role of serological testing, especially, in light of the new classification of Interstitial Pneumonia with Autoimmune Features (IPAF) and discuss the evidence for therapies targeted at idiopathic and immune‐related pulmonary fibrosis.

## Graphical abstract

Interstitial lung diseases (ILD) are a large group of conditions causing morbidity and mortality in all age groups. In addition to highlighting the benefits of a multidisciplinary diagnoses, we review developments in ILD diagnostics, especially around autoimmune disease and current evidence for newer therapeutics.

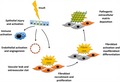

## Introduction

Interstitial lung disease (ILD) is an umbrella term for a large group of over 200 pulmonary disorders characterised by inflammation and/or fibrosis of the pulmonary interstitium with heterogeneous causes, clinical course and treatments. While some ILDs have an identifiable trigger with occupational, environmental or medication exposure and others are strongly associated with systemic disease, in particular the connective tissue diseases (CTD), for many their origin is unknown and the term ‘idiopathic’ is applied.^1^, ^2^, ^3^ The largest group of these are the idiopathic interstitial pneumonias (IIP).^4^, ^5^

Early work on understanding these diseases was hampered by a lack of shared criteria for diagnosis and treatment trials which were often small and involved mixed groups of patients. More recently, professional societies across the world have published joint position papers on disease classification and approach to treatment which continue to evolve with inclusion of new entities and evolving criteria for diagnosis.^1^, ^3^, ^6^, ^7^ However, despite these improvements in classification, there is still a substantial overlap in the clinical, radiological and pathological features across the different ILD entities, making an accurate diagnosis challenging.^8^ This particularly applies to the IIPs, CTD‐ILDs and a group defined more recently as idiopathic pneumonia with autoimmune features (IPAF) which overlaps with, but appears separate to, both the idiopathic and CTD‐associated ILDs.

For the idiopathic interstitial pneumonias (IIP), there are no identifiable triggers, although genetic predisposition is starting to be documented.^1^, ^3^ The commonest radiological and pathological patterns are usual interstitial pneumonitis (UIP – giving a diagnosis of idiopathic pulmonary fibrosis (IPF)), non‐specific interstitial pneumonitis (NSIP) and organising pneumonitis (OP). CTD‐ILDs are commonly seen in a number of CTDs such as rheumatoid arthritis and systemic sclerosis where the radiological and pathological patterns similarly include UIP, NSIP and OP. In IPAF, patients may have a range of clinical (Raynauds, arthritis), serological (autoantibodies) and morphological (NSIP and OP) features which overlap with the idiopathic and CTD groups but do not meet the criteria for either of the conditions.

As many ILDs are progressive with a poor prognosis, identifying the correct aetiology is clinically important, influencing treatment choice and understanding of prognosis. Additionally, accurate phenotyping informs disease epidemiology, provide insights into pathophysiology of disease and facilitates the design of clinical studies.^2^

Treatment options for patients have increased with the availability of anti‐fibrotic therapy, in addition to an increasing range of immunosuppressive agents clinicians can choose from.^5^ It is early days in terms of available data with the use of anti‐fibrotics in any interstitial disease other than IPF, but there is good evidence immunosuppressive therapy can do harm in some forms of ILD.

This review will explore the latest research around making an accurate diagnosis, in the overlapping entities of IIP, CTD‐ILD and IPAF, and will review the implications of recent data on serological testing for connective tissue diseases and the early clinical data on genetics behind ILD. It will explore the implications of having two approaches to therapy, namely with anti‐fibrotic or immunosuppressive therapy and the current evidence behind helping to choose the best for patients.

## Classification

### Idiopathic interstitial pneumonias and IPF

**Figure 1:**
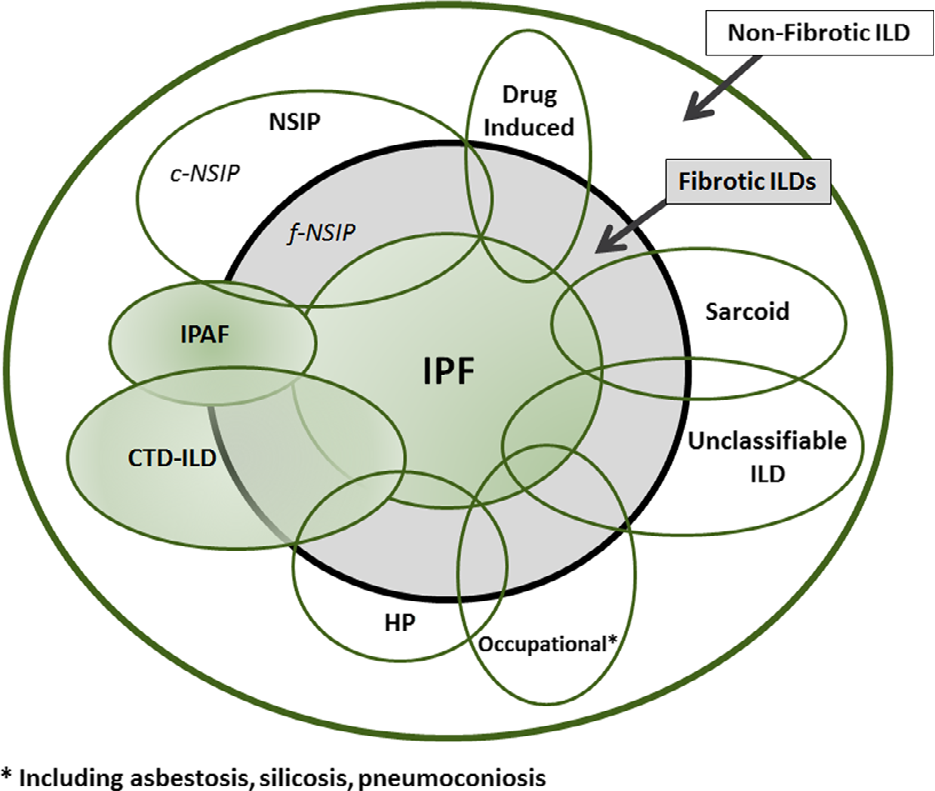
Disease overlap between idiopathic pulmonary fibrosis (IPF) and other interstitial lung diseases (ILD). The shaded conditions represent the focus of this paper. The size of the ovals reflects the approximate prevalence of individual diseases. IPAF: interstitial pneumonitis with autoimmune features; c‐NSIP: cellular fibrotic non‐specific interstitial pneumonia; CTD: connective tissue disease; f‐NSIP: fibrotic non‐specific interstitial pneumonia; HP: hypersensitivity pneumonitis. (adapted from Wells *et al*.^75^).

Idiopathic interstitial pneumonias, as shown in Figure 1, include a range of diseases which are common (such as IPF and NSIP) through to rare (such as lymphocytic interstitial pneumonia and idiopathic pleuroparenchymal fibroelastosis). We will focus on IPF, as it is the most common form and has been the most intensely studied of IIPs with well‐defined criteria for diagnosis, and when clinical and radiological findings are classical, a lung biopsy is not required. It is more common in males with patients typically presenting in the 6th–7th decade with insidious exertional breathlessness and cough. Incidence is increasing with estimated 3‐9 new cases per 100 000 per year in Europe and North America each year.^4^, ^9^, ^10^ Clinical findings of basal crackles and clubbing are common. It is a progressive condition with a poor prognosis with a median survival of only 2 to 5 years after diagnosis. The pathogenesis is thought to involve epithelial cell injury with aberrant repair. A key finding both radiologically and on histopathology is the pathological pattern of usual interstitial pneumonia (UIP).^9^ Radiological changes are typically bilateral with basal and subpleural predominant reticular infiltrates with architectural distortion associated with traction bronchiectasis and honeycombing. Pure ground‐glass changes are discordant with the diagnosis. Pathologically, the findings are of subpleural and paraseptal dense fibrosis, with remodelling and honeycomb fibrosis, fibroblastic foci and patchy lung involvement that appears heterogenous in terms of disease stage. Specific findings such as granuloma or organising pneumonia should be absent. A key step in diagnosis is to exclude other overlapping conditions, and the new frameworks use the terms ‘definite’, ‘probable’, ‘indeterminate’ and ‘other diagnosis’ to categorise certainty of diagnosis and guide when a biopsy is recommended.^6^, ^9^ They take into account clinical, serological, radiological and where available pathological findings. Increasing age and average total fibrosis CT score have, for example, in probable UIP, where there is an absence of honeycombing, shown correlation as markers to predict IPF findings on biopsy.^11^ This may prove clinically meaningful to those patients deemed unfit for biopsy. Treatment has universally been dismal until the last decade with the demonstration that two anti‐fibrotic medications, pirfenidone and nintedanib, can slow the rate of progression and decrease exacerbations.^5^

### CTD‐associated ILD

Many of the connective tissue disorders (including rheumatoid arthritis, polymyositis/dermatomyositis, systemic lupus erythematosus, systemic sclerosis and mixed connective tissue disease) are associated with interstitial disease. The radiological and pathological findings are the same as with the many of the idiopathic forms of interstitial pneumonias with the commonest disease patterns being UIP, fibrotic NSIP and OP with variation across the CTDs being the most prevalent. The interstitial disease may be the most important determinant of prognosis, and as it can precede or be the predominant feature in some of these conditions,^1^, ^12^ serology may play an important part early in disease to differentiate CTD‐ILD from IIP. Accurate diagnosis has significant implications for both prognosis and treatment.

For certain patterns of disease, for example, UIP, patients with CTD‐ILD generally have a better prognosis than patients with IPF with a slower progression of disease, with the notable exception of UIP in rheumatoid arthritis‐associated ILD.^13^ The current approach to treatment in CTD‐ILDs involves immunosuppression, but it is not yet known, for example, when a UIP pattern is present, whether this should be changed to anti‐fibrotics as is used in IPF.^7^, ^14^

### Interstitial pneumonia with autoimmune features (IPAF)

In addition to those patients who meet the criteria for diagnosis of a CTD, a significant number of patients with ILD have clinical features suggestive of an underlying autoimmune process, but yet do not meet the defined diagnostic criteria for CTD. These patients have been given the designation of interstitial pneumonia with autoimmune features (IPAF). In order to provide a uniform criterion to allow comparative studies of such patients, the European Respiratory Society and American Thoracic society proposed the nomenclature of IPAF for these patients and recommended classification criteria.^7^ These criteria incorporate three separate domains including clinical (such as Raynaud’s phenomenon, inflammatory arthritis), morphological (including radiological and histological patterns) and serological (Table 1). This grouping remains a problem and the term can be thought of as useful, in particular in the research setting, rather than a firm disease entity. A recent review comparing four recent retrospective studies showed that populations were heterogeneous in terms of features and outcomes, and that there was variable involvement of rheumatologists in the diagnostic process and inconsistent terminology was used.^15^ Special points of concern were around the inclusion of early CTD patients, especially anti‐synthetase syndrome (potentially delaying treatment), and limited the availability of extended panels of antibodies.^15^, ^16^ Clearly, this is a group of patients where the criteria for diagnosis will continue to evolve.

**Table 1:** Criteria for interstitial pneumonia with autoimmune features (IPAF)

Criteria for Interstitial Pneumonia with Autoimmune Features (IPAF)^†^		
Presence of interstitial pneumonia by HRCT or surgical lung biopsy Exclusion of alternative aetiologies Does not meet criteria for a defined CTD At least one feature from 2 of the 3 following domains		
A. Clinical domain	B. Serologic domain	C. Morphological domain
Raynauds phenomenon Palmar telangiectasia Distal digital fissuring (i.e. ‘‘mechanics hands’’) Distal digital tip ulceration Inflammatory arthritis or polyarticular morning joint stiffness> 60 min Unexplained digital oedema Unexplained fixed rash on the digital extensor surfaces (Gottron sign)	ANA titre ≥ 1:320, diffuse, speckled or homogeneous patterns or ANA nucleolar pattern (any titre) or ANA centromere pattern (any titre) RF> 2 x ULN Anti‐CCP Anti‐dsDNA Anti‐Ro antibodies (SS‐A) Anti‐La antibodies (SS‐B) Antiribonucleoprotein Anti‐Smith antigen Antitopoisomerase (Scl‐70) Anti‐tRNA synthetase (e.g. Jo‐1, PL‐7, PL‐12) Anti‐PM‐Scl Anti‐MDA‐5	*Suggestive radiological patterns:* NSIP pattern OP pattern Mixed NSIP/OP pattern LIP pattern *Histopathology features by surgical lung biopsy:* NSIP OP NSIP with OP overlap LIP Interstitial lymphoid aggregates with germinal centres Diffuse lymphoplasmacytic infiltration (with or without lymphoid follicles) *Multicompartment involvement (in addition to IP)* Unexplained pleural effusion or thickening Unexplained pericardial effusion or thickening Unexplained intrinsic airways disease (by PFT, HRCT or pathology) Unexplained pulmonary vasculopathy

ANA, antinuclear antibody; CCP, cyclic citrullinated peptide; CTD, connective tissue disease; dsDNA, double‐stranded deoxyribonucleic acid; HRCT, high‐resolution computed tomography; LIP, lymphoid interstitial pneumonia; MDA, melanoma differentiation‐associated; NSIP, non‐specific interstitial pneumonia; OP, organising pneumonia; PFT, pulmonary function tests; PM‐Scl, polymyositis/systemic scleroderma; RF, rheumatoid factor; tRNA, transfer RNA; ULN, upper limit of normal.

^†^ Reproduced with permission of the © ERS 2019: European Respiratory Journal 46 (4) 976‐987; https://doi.org/10.1183/13993003.00150-2015 Published 30 September 2015.

## Diagnostics

A multidisciplinary approach to diagnosis with experts in respiratory medicine, radiology, immunology, rheumatology and pathology reviewing findings at a multidisciplinary meeting (MDM) has been shown to increase diagnostic confidence and inter‐observer agreement.^8^ MDMs in ILD encourage comprehensive clinical, serological and radiological workup, adherence to up‐to‐date published diagnostic criteria and should allow access to ILD expertise beyond the tertiary setting, for a large group of patients and their physicians in the community. MDMs should have both a diagnostic role and an advisory role and, in addition to new cases, reassessment of cases based on additional clinical information (e.g. response to therapy, rate of disease progression) should occur, particularly were atypical. In one Australian study, the introduction of the MDM resulted in changes to specific ILD diagnosis in 53% of cases with an increase in CTD‐ILD diagnosis from 10 to 21%.^17^ Conversely, evidence stipulating change in diagnoses after serial MDM discussions is lacking but may become available as the momentum for ILD registries continues. Discordant results, even in the most expert of centres, remain problematic, particularly as the constitution of worldwide MDTs does vary and access to treatment is dependent on the diagnosis.^18^

An accurate diagnosis starts with a comprehensive clinical assessment. A detailed history should include symptoms, disease course, presence of associated diseases, in particular of connective tissue disorders, history of occupation, hobbies or environmental exposures, medications and family history. Typical signs sought include lung crackles, clubbing and any signs of a connective tissue disease with joint, muscle or skin involvement. Lung function classically shows reduced lung volumes and diffusing capacity with desaturation on walking.

### Radiological

Plain x‐rays are often performed during the initial assessment but provide limited information and the key clinical tool is the high‐resolution CT (HRCT) scan. In IPF, the classical HRCT finding of UIP pattern in the setting of a consistent clinical picture equate to a high probability of IPF, and obviates the need for a lung biopsy. The recently published ATS/ERS/JRS/ALAT Clinical Practice Guideline 2018,^4^ The Diagnosis of Idiopathic Pulmonary Fibrosis, provides a guide from the technical aspects of performing a HRCT (such as prone imaging, inspiratory and expiratory high‐resolution slices) through to interpretation of the images enabling MDMs to categorise the HRCT as ‘definite’ UIP, ‘probable’ UIP, ‘indeterminate’ and an ‘alternate diagnosis’, which correlates to the likelihood of IPF in the absence of alternatives based on clinical, serological or other testing. However, studies have shown moderate inter‐observer agreement by radiologists for HRCT, even between highly experienced radiologists.^19^ Furthermore, it is important to note that it is not uncommon to see combination patterns of fibrosis, particularly in CTD‐ILD or drug‐induced ILD, highlighting the barriers to relying on radiology alone.

### Histopathology

The challenge in histopathology to make an accurate diagnosis is obtaining a sample of sufficient size. Transbronchial biopsies (TBB) are mostly insufficient, except in a few circumstances (sarcoidosis, organising pneumonia), and therefore surgical lung biopsy (SLB) is required. This carries a significant mortality risk of 3.5% (95% CI, 2.8–4.3%), which is lower in the elective setting but higher in a deteriorating patient or one with co‐morbidities.^20^ In one American study, average mortality for elective SLB was 1.7% versus 16% for non‐elective noting that severity of hypoxia directly correlated with worse outcomes.^21^ The interpretation of the biopsy, as with radiology, requires a pathologist with a special interest in ILD and findings to allow categorisation similar to HRCT interpretation with definite, probable and indeterminate for UIP or an alternate diagnosis according to defined guidelines. However, again as with radiology, studies have shown poor inter‐observer agreement between pathologists, even when biopsies are read by expert thoracic histopathologists.^22^ As is often the case, SLB is often reserved for those difficult or atypical cases offering one reason behind discordant results. Additionally, the exact timing of biopsy (early versus late) in the disease is not clear given the heterogeneity of disease patterns and prognosis. It could be argued that early diagnosis of IPF, for example, would enable access to anti‐fibrotic therapy early and significantly (positively) impact median survival. This will become clearer as longitudinal data on anti‐fibrotic use becomes available. Of course, this decision must acknowledge both the mortality risk and the ILD exacerbation risk of the procedure.

As a consequence of the morbidity and mortality associated with SLBs, there has been growing interest in transbronchial lung cryobiopsy (TBLC) which appears to provide a good yield of tissue with a more acceptable risk (mainly from bleeding), although performance may be less acceptable in non‐expert centres. In a recent study where paired TBLC and SLB were taken in the same patient and read by a blinded expert pathologist, the TBLC and SLB were poorly concordant, with only 38% agreement (95%CI: 18‐62%) and the SLB carrying more weight in making the final diagnosis. It is not quite clear as to where TBLC fits in the investigation of patients with interstitial disease,^23^ but a large, randomised controlled trial in Australia (COLDICE) has just finished recruiting ILD patients for sequential TBLC and video‐assisted thorascopic SLB and may answer this very question.

Early work on the use of genomic classifier or gene expression signatures on multiple TBB samples from a patient can increase the diagnostic yield in differentiating UIP from non‐UIP better than TBB alone.^10^ This has yet to be applied in a prospective manner against the gold standard of a SLB to confirm its clinical credibility. Bronchoalveolar lavage, a safe procedure, has been studied for decades and was previously viewed as providing a ‘liquid’ lung biopsy. Its role is more limited these days and is not performed routinely. However, the finding of lymphocytosis in the setting of appropriate clinical and radiological findings greatly increases the likelihood of the presence of hypersensitivity pneumonitis.^24^

### Autoantibody testing

Diagnosis of patients with connective tissue disease ILD (CTD‐ILD) is often challenging, but in the presence of interstitial lung disease, it is important to differentiate CTD‐ILD from idiopathic forms or from IPAF as it has implications for prognosis and treatment, with CTD‐ILD generally having a better prognosis than patients with IIP.^14^

Detection of specific autoantibodies in serum plays a key role in the diagnosis of CTD‐ILD, but there remains a paucity of clinical evidence on which of them can guide routine clinical practice. Many guidelines recommended testing for a narrow panel of autoantibodies only, with additional testing performed according to relevant clinical signs and symptoms suggestive of an underlying CTD.^4^ ILD may be associated with all CTDs, and whilst this is most commonly seen in established CTD, patients may present with ILD as the initial feature of their CTD prior to subsequent development of other CTD clinical features, or may present with isolated ILD as the sole clinical manifestation of a CTD.^25^, ^26^ Detection of autoantibodies may reveal previous undiagnosed CTD in patients, even when seen in specialist ILD clinics, and restricting serological testing according to clinical features will inadvertently miss patients with CTD‐ILD.^27^ In a recent study of 80 undifferentiated ILD patients where retrospective autoantibody testing was performed to a comprehensive autoantibody panel, the ILD diagnosis was able to be reclassified in 6 of 80 (7.5%) cases to CTD‐ILD.^28^ The clinical features or basic autoantibody tests alone (antinuclear antibodies, rheumatoid factor positivity) did not accurately predict the presence of myositis antibodies or ANCAs and recommendations for broad screen were made (Table 2). Prospective studies to assess the economic benefit of this expanded repertoire of serological screening tests are, however, still needed. In addition, the need for serial testing of autoantibodies from ILD patients remains unclear, as some patients can develop antibody positivity after the diagnosis of ILD. A Chinese study of 1044 CTD‐ILD patients showed seroconversion, with 262 (25.1%) patients who were antibody negative at initial presentation developing autoantibodies during follow‐up.^29^ The ideal frequency of serial monitoring and in whom this should be targeted remains unknown.

**Table 2:** Extended panel for autoimmune serology in diagnostic assessment of ILD suggested at initial assessment suggested by Stevenson *et al*.^28^

Autoantibody	Disease associations
ANA	SLE, SjS, SSc, PM, DM, MCTD
ENA including:	
SS‐A	SjS, SLE
SS‐B	SjS, SLE
Ro52	SjS, SLE, PM, DM
Ribosomal P	SLE
Histones	Drug‐induced SLE, RA
Scl70	SSc
anti‐Sm	SLE
anti‐RNP	MCTD, SLE, SSc
dsDNA	SLE
Rheumatoid factor	RA, SjS
Anti‐CCP antibody	RA
Myositis‐specific antibodies (Jo‐1, PL‐7, PL‐12, EJ, OJ, KS, SRP, Mi2, NXP2, TIF1γ)	PM, DM, anti‐synthetase syndromes
Myositis‐associated antibodies (Ku, PMScl75, PMScl100)	PM, DM, SSc, SSc‐PM overlap, SLE
ANCA including MPO and PR3	ANCA‐associated vasculitis

DM, dermatomyositis; MCTD, mixed connective tissue disease; PM, polymyositis; RA, rheumatoid arthritis; SjS, Sjogrens syndrome; SLE, systemic lupus erythematosus; SSc, systemic sclerosis.

Further to the diagnosis of CTD‐ILD, autoantibody screening plays a role in the newer entity IPAF where patients have ILD and features suggestive of an underlying autoimmune process, but yet do not meet the defined diagnostic criteria for CTD. Serology is the key to the definition, along with clinical and morphological findings (Table 1). Given the low specificity of ANAs and rheumatoid factor, only high titres (ANA ≥ 1:320 and RF level> 2 times upper limit of normal) are included along with more specific antibodies including extractable nuclear antigens (ENA), anti‐CCP and myositis antibodies. There are a number of limitations with these criteria in their current form. High weight is placed upon serological testing, such that patients with UIP, non‐specific interstitial pneumonia (NSIP), lymphocytic interstitial pneumonia (LIP) or organising pneumonia meet the IPAF diagnostic criteria in the presence of a single seropositive result only.

A significant problem remains the lack of standardisation of laboratory testing. For example, assessment of ANA testing shows significant variability in reported levels for identical samples both within and between laboratories.^30^, ^31^ The criteria also include antibodies with a high specificity for other CTDs such that high levels of these autoantibodies (e.g. dsDNA for systemic lupus erythematosus or rheumatoid factor and anti‐CCP for rheumatoid arthritis) have extremely high probabilities of eventually meeting diagnostic criteria for CTD and needing to be reclassified as CTD‐ILD rather than IPAF.

Exclusion of ANCA antibodies may exclude a large group of potential IPAF patients. In a Japanese study of ILD patients, 26 patients were identified with MPO‐ANCA of whom 16 were positive at diagnosis and 10 seroconverted to positive during follow‐up. Of these patients, only 9 (35%) progressed to develop an ANCA‐positive vasculitis over a five‐year observation period (in all cases microscopic polyangitis).^32^ Another review of 92 ANCA‐positive patients from 5 IPF cohorts revealed a concurrent vasculitis was diagnosed in 35.8% (31 cases with microscopic polyangiitis, 2 cases with granulomatous polyangiitis), a delayed diagnosis of ANCA‐associated vasculitis in 17.4% (16 cases with MPA) with almost half (46.8%) having IPF with ANCA positivity but no evidence of ANCA‐associated vasculitis.^33^ In contrast, the recommended IPAF cohort may contain a number of patients with hypomyopathic or amyopathic myositis, as most myositis‐specific antibodies have remained excluded from current diagnostic criteria for dermatomyositis and polymyositis, including the most recent EULAR/ARC guidelines.^34^ Myositis autoantibodies have been detected in 6.6–38% of ILD cases depending on the study population, methods of antibody detection and panels of antibodies assessed.^28^, ^35^, ^36^ A multicentre study using an ELISA system for detection of six anti‐synthetase antibodies (Jo‐1, EJ, KS, OJ, PL‐7, PL‐12) identified antibodies in 10.7% of IPF patients.^37^ Cases of ILD in the absence of diagnostic features of dermatomyositis or clinically amyopathic dermatomyositis have been described for most of the myositis antibodies including MDA‐5, PL‐7, PL‐12, OJ and EJ. Exclusion of patients with these antibodies may help harmonise the IPAF cohort. However, overall, the development of these IPAF criteria represents a significant step in harmonising data to allow comparison across studies. Given that IPAF encompasses a diverse group of patients including patients with phenotypes such as myositis‐like features, it will be important that these studies are powered to compare these phenotypes and to identify whether there are even subgroups of IPAF, which may affect prognosis or therapeutic decisions.

### Genetic studies

Genetic studies are an important adjunct to clinical assessment in a number of areas of medicine including in neurodegenerative diseases, where they aid diagnosis, and in oncology, where they may influence the choice of immunotherapy. Genetic studies in interstitial lung diseases have mainly been limited to familial pulmonary fibrosis (FPF) patients and patients with the sporadic form with IPF.^38^, ^39^ Mutations in a number of genes implicated in disease susceptibility have been identified (Table 3). However, in one FPF cohort, identifiable genes explained only one‐third of the disease.^40^ The commonest genes found in both FPF and sporadic IPF are *MUC5B,* which encodes a member of the mucin family of proteins; *TOLLIP,* which encodes a toll‐inhibiting protein which inhibits toll receptor signalling; telomere‐related genes, of which *TERT* encoding telomere reverse transcriptase is the most common; and *SP‐C,* encoding hydrophobic surfactant protein C essential for lung function and homoeostasis. 40, 41 In telomere‐related genetic mutations, there is poor genotype‐ILD phenotype correlation across patients. In a study of 115 ILD patients with telomere‐related mutations, multidisciplinary diagnosis was of IPF in 46%; unclassifiable in 20%; chronic hypersensitivity pneumonitis in 12%; pleuroparenchymal fibroelastosis in 10%; interstitial pneumonia with autoimmune features (IPAF) in 7%; idiopathic interstitial pneumonia in 4%; and other connective tissue disease‐related ILD in 3%.^39^ Presumably, environmental factors along with other genetic factors interact to lead to a particular penetrance and clinical phenotype, although the presence of telomere mutations did predict uniformly progressive disease. Interestingly, studies have shown the same genes, in particular MUC5b is associated with predisposition to rheumatoid arthritis‐associated ILD (RA‐ILD), suggesting some shared pathogenesis between IPF and RA‐ILD.^42^ Genetic studies in IPF or FPF patients are gaining interest in lung transplant workup where it has been shown patients with short telomeres have a higher rate of complications.^43^ Additionally, short telomeres in the donor may also relate to worse outcomes.^44^ How genetic studies can help us in terms of diagnosis or treatment will not be known for some time and will require extensive research.

**Table 3:** Genetic mutations in pulmonary fibrosis

Genetic mutations in pulmonary fibrosis	Associated conditions
Telomerase and telomere‐related genes	
Dyskerin *(DKC1)*	IPF
Poly(A)‐specific ribonuclease *(PARN)*	IPF, IPAF, CHP
Regulator of telomere elongation helicase (*RTEL1*)	IPF, IPAF, CHP, PPFE
Telomerase reverse transcriptase (*TERT*)	IPF, IPAF, HP, CTD‐ILD, PPFE, NSIP, DIP, CPFE
Telomerase RNA component *(TERC)*	IPF, HP, CTD‐ILD, PPFE, CPFE
Telomere interacting factor 2 *(TINF2)*	IPF
Surfactant protein‐related genes	
ATP‐binding cassette‐type 3 *(ABCA3)*	IPF, CPFE
Surfactant protein C *(SFTPC)*	IPF, CPFE
Immune function‐related genes	
Human Leucocyte antigen, DRB1 *(HLA‐DRB1)*	IPF, HP, CTD‐ILD
Interleukin 8 *(IL8)*	IPF
Toll‐interacting protein *(TOLLIP)*	IPF
Toll‐like receptor 3 *(TLR3)*	IPF
Transforming growth factor β‐1 *(TGFB-β1)*	IPF
Others genes	
Family with sequence similarity 13, member A *(FAM13A)*	IPF
Mucin 5B *(MUC5B)*	IPF, CHP, RA‐ILD

CPFE, combined pulmonary fibrosis and emphysema; CTD‐ILD, connective tissue diseased–interstitial lung disease, IPF, idiopathic pulmonary fibrosis; IPAF, interstitial pneumonia with autoimmune features; HP, hypersensitivity pneumonitis; NSIP, non‐specifc interstitial pneumonitis; PPFE, pleuroparenchymal fibroelastosis; RA‐ILD, rheumatoid arthritis‐associated interstitial lung disease.

## Treatment of ILD

The treatment landscape in ILD has changed dramatically in the last decade with available anti‐fibrotics, and whilst a cure remains elusive, meaningful gains in longevity have been achieved.^45^ The two main approaches in terms of disease‐modifying drugs will be outlined below with immunosuppressive and anti‐fibrotic therapies.

**Figure 2:**
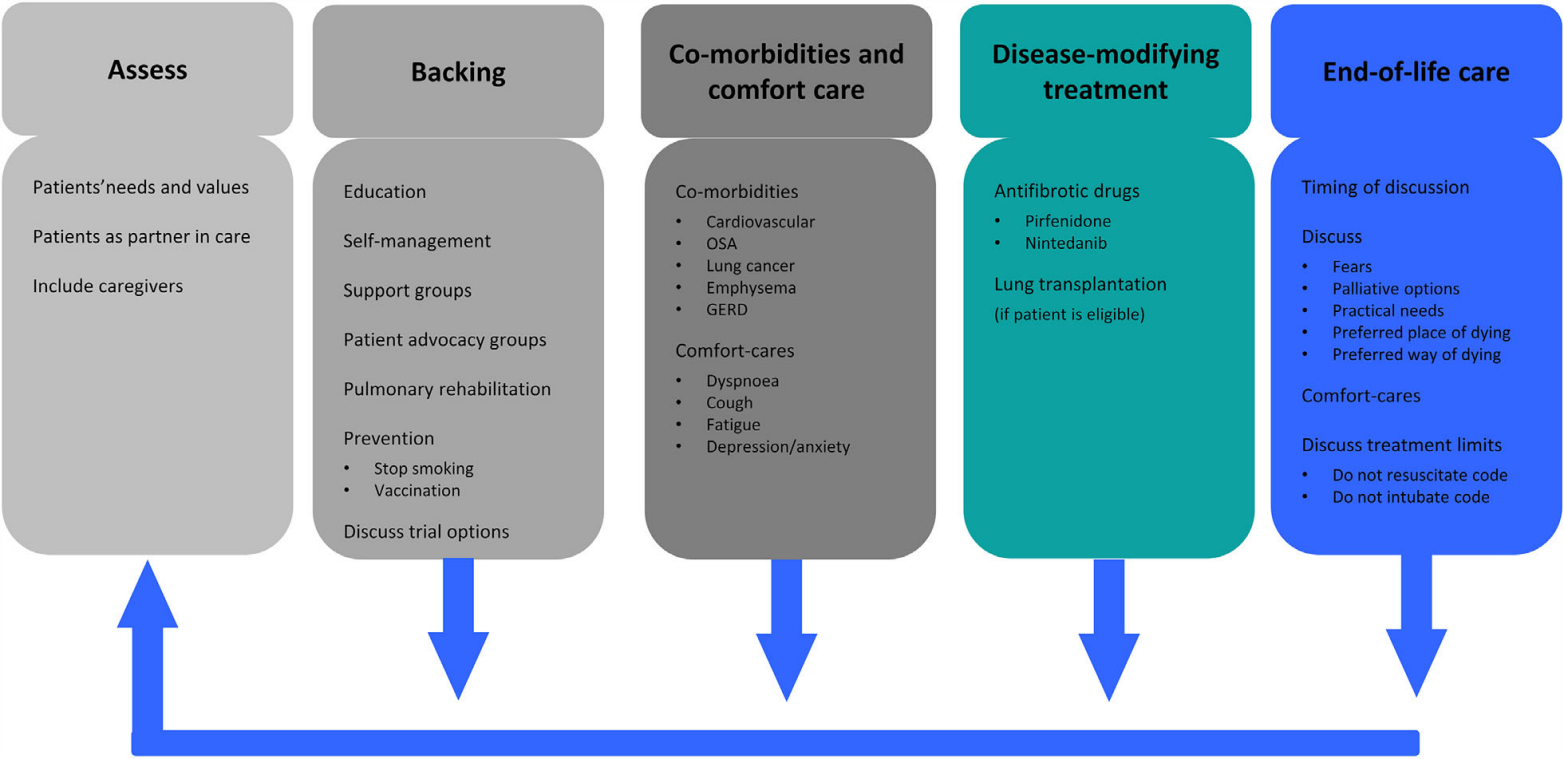
ABCDE of idiopathic pulmonary fibrosis care. GERD, gastro‐oesophageal reflux disease; OSA, obstructive sleep apnoea (reproduced from van Manen *et al.* 46 with permission).

Treatment focus in all patients should, however, start with a patient‐centred approach on symptoms and understanding not just disease‐modifying drugs. Van Manen *et al.* propose an ABCDE approach with **A**ssessment of patients’ needs and values; **B**acking with education; **C**o‐morbidities and comfort care; **D**isease‐modifying treatment and **E**nd‐of‐life care (Figure 2).^46^ Oxygen therapy in ILD lacks robust data as highlighted in a recent meta‐analysis reviewing the impact of oxygen on dyspnoea, quality of life, exercise capacity and mortality in ILD patients.^47^ Whilst improvement in exercise capacity was observed, no demonstrated mortality benefit was seen in any of the referenced studies. Pulmonary rehabilitation has demonstrated benefit in ILD patients, albeit that the benefit is not sustained once exercise programmes cease.^5^, ^48^ Lung transplantation may also be considered in ILD, although this poses challenges as many patients are older with more co‐morbidities than other transplant cohorts. Unfortunately, many patients with scleroderma‐associated CTD‐ILD are not suitable because of the association of poorer outcomes with co‐existent reflux and poor wound healing. For many, palliative care is essential but should be introduced early in the disease process with an emphasis on symptomatic care.^49^

**Figure 3:**
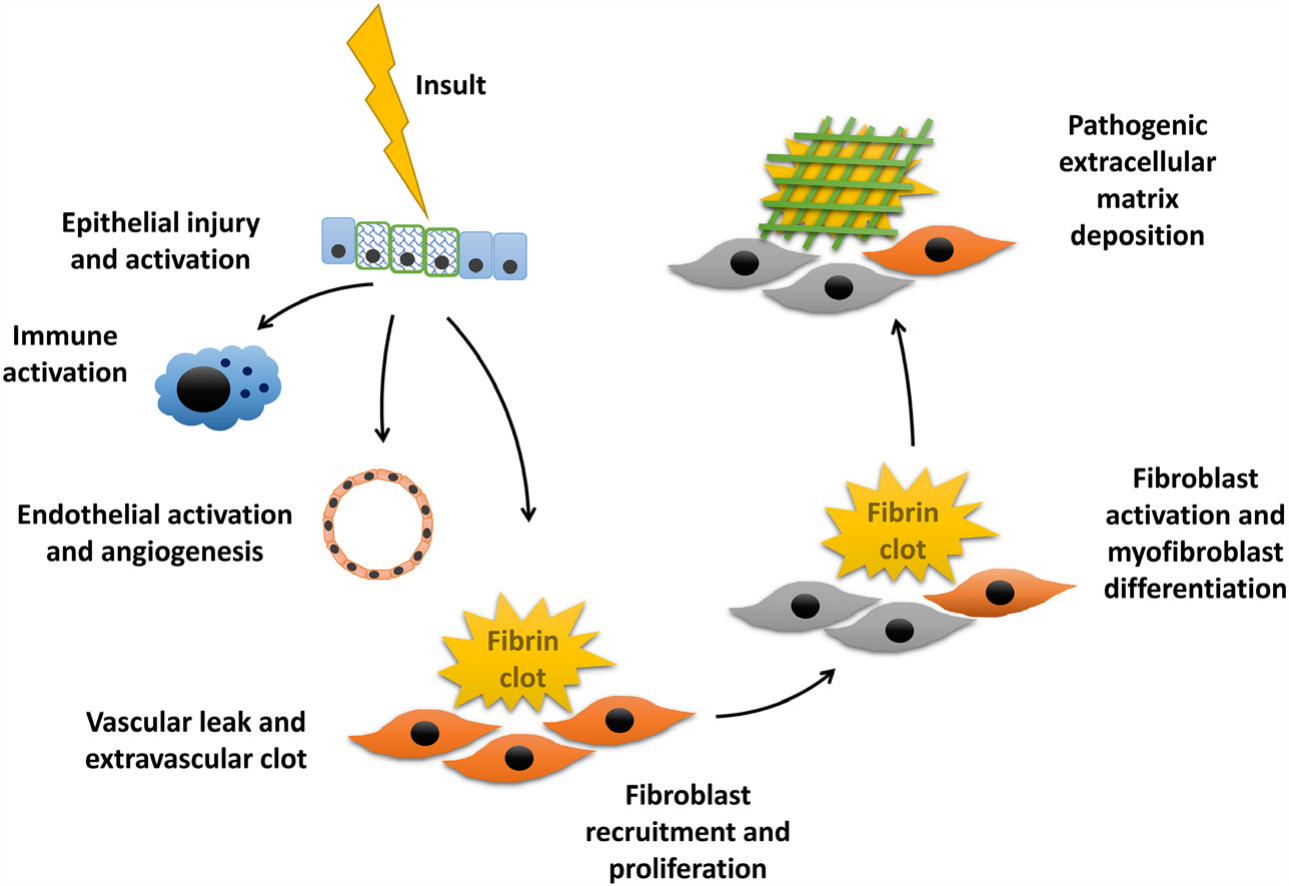
Schematic diagram of sequence of profibrotic processes implicated in the current understanding of IPF pathogenesis which results in fibrosis rather than normal repair. All of these stages are targets for potential therapeutic intervention.

In terms of therapy aimed to modify the course of disease, the original working model describing the pathogenesis of interstitial lung diseases was that inflammation preceded and caused fibrosis, suggesting patients would benefit from immunosuppression, in particular early in the disease when it was thought inflammation was greatest. This was indirectly supported by retrospective case studies, and it was not until around the year 2000 that guidelines acknowledged the poor evidence to support such treatment and 2012 when a placebo‐controlled trial was stopped early because of a higher mortality with prednisolone and azathioprine and N‐acetyl cysteine.^50^ Despite this insight, it has taken years for practice to change as the universally poor prognosis, in particular with IPF, drove a desire to be proactive with treatment. Subsequent research led to a model of injury followed by aberrant would repair, as outlined in Figure 3, which has now turned the focus away from inflammation to fibrosis and alternate treatment options.

A major stumbling block remains the limited availability of robust randomised control trial data outside the IPF cohort and the heterogeneity of both disease subtypes and disease progression across and within disease groups (such as RA‐ILD or the IIPs). An alternate approach to choosing treatment has been proposed where a patient’s disease is classified by clinical phenotype which captures the rate of progression,^51^ and under these circumstances drugs aimed at preventing progressive fibrosis are used. Several studies are underway, where, for example, those with any CTD with a UIP or fibrotic NSIP (‘progressive’) pattern but not OP or cellular NSIP pattern (generally better prognosis without fibrosis) will receive anti‐fibrotic treatment, which so far has only been studied in IPF. The results will potentially significantly change the treatment approach and are keenly awaited.^52^, ^53^

### Anti‐fibrotic therapy

Trials of anti‐fibrotics arose from basic research in animal models demonstrating the underlying fibrotic pathogenesis including an appreciation of aberrant wound healing and deposition of collagen in the extracellular matrix as key features. Presently, there are only two licensed drugs in the treatment of IPF: pirfenidone and nintedanib. Pirfenidone (5‐methyl‐1‐phenyl‐2‐[1H]‐pyridone) is a synthetic oral drug shown to inhibit collagen synthesis and fibroblastic proliferation in animal models through regulation of transforming growth factor (TGF) β and tumor necrosis factor (TNF) α.^54^ Nintedanib, in contrast, is a tyrosine kinase inhibitor with action against platelet‐derived growth factor receptors (PDGF), fibroblast growth factor receptors (FGF) and vascular endothelial growth factor receptors (VEGF), all of which are involved in signalling pathways linked to the pathogenesis of pulmonary fibrosis.^55^

In Australia, patients can access either of these drugs provided they have an MDM diagnosis of IPF, forced expiratory volume in 1 second (FEV1)/forced vital capacity (FVC) ≥ 0.7, FVC ≥ 50% and diffusing capacity of carbon monoxide (DLCO) ≥ 30% and, importantly, must not have interstitial lung disease because of other known causes including domestic and occupational environmental exposures, connective tissue disease or drug toxicity.

The CAPACITY trials were two concurrent multinational randomised control trials assessing the efficacy of pirfenidone compared to placebo over 72 weeks in patients aged 40–80 years with an ILD MDT diagnosis of IPF. Inclusion criteria included predicted FVC ≥ 50% (but ≤ 90%), predicted DLCO of ≥ 35% and 6‐min walk test (6MWT) distance of at least 150 m. The primary end point of lower percentage decline in FVC was significantly favorable in the pirfenidone group.^56^, ^57^ Interestingly, 20% of the pirfenidone group still had a decline in FVC of > 10% (diagnostically and clinically meaningful in ILD), perhaps reflecting other signalling pathways at play. Gastrointestinal upset with nausea, photosensitivity and rash were predominant adverse effects. The further phase III ASCEND trial in 2014 showed a 47.9% reduction compared to placebo in decline ≥ 10% in FVC or death.^58^

INPULSIS‐1 and −2 were, again, two concurrent multinational double‐blind randomised controlled trials conducted over 52 weeks comparing nintedanib with placebo. Inclusion criteria were patients aged ≥ 40 years with a diagnosis of IPF (based on radiology expert opinion) established within five years before randomisation, FVC ≥ 50% predicted and DLCO of 30‐79% predicted. Like pirfenidone patients, this group demonstrated a statistically significant adjusted annual rate of change in FVC of −114.7 mL with nintedanib versus −239.9 mL with placebo.^59^ An improvement in time to first exacerbation was reported in INPULSIS‐2 (hazard ratio, 0.38; 95% CI, 0.19–0.77; *P* = 0.005), but this was not replicated in INPULSIS‐1. Diarrhoea was the most common adverse event with a high prevalence (rate of 62.4%) but only accounting for complete discontinuation in just under 5% of patients.^60^

Post hoc analysis in both pirfenidone and nintedanib trials demonstrated that the rate of decline regardless of predicted FVC starting point (i.e. mild, moderate or severe disease) is similar across all groups prompting health care professionals to think about introducing anti‐fibrotic therapy sooner and perhaps before a patient is symptomatic. This clearly requires further evaluation with appropriate cost‐benefit analysis. To date, there have been no head‐to‐head studies between anti‐fibrotic therapies and so presently choice of anti‐fibrotic lies with the physician and the patient. From trial data, we have derived certain risk criteria – patients with intended high sun exposure would favor nintedanib because of photosensitivity with pirfenidone and patients on anti‐coagulants favor pirfenidone because of increased bleeding risk with nintedanib for example. Other contributing factors include pill burden, history of ischaemic heart disease and gastro‐oesophageal reflux disease to name a few.^61^ Additionally, as will be detailed, data on their role in the treatment of other interstitial diseases and in combination with other drugs are urgently required.

### Immunosuppression

Prednisolone has been commercially available from the mid‐50s and has been the mainstay for the management of many immune or inflammatory conditions. It is potent, very broad in action and unfortunately associated with many side effects. Historically, it alone or in combination with azathioprine was the treatment given for IPF and many other forms of ILD. It was not until relatively recently definitive evidence became available demonstrating the potential harm of immunosuppression in IPF. The azathioprine, prednisolone and N‐acetylcysteine arm of the PANTHER‐IPF trial was discontinued early because of an increased risk of hospitalisation and death compared to the placebo and N‐acetylcysteine alone arms.^50^ This was the first high‐level evidence for what many clinicians already suspected and subsequently led to recommendations to avoid immunosuppression in IPF. Questions, however, remain as to whether alternative immunosuppressive therapies, either alone or on combination with anti‐fibrotics, may be beneficial as the current studies are small and retrospective.^62^, ^63^

In contrast to IPF, immunosuppression is still first‐line non‐licensed therapy in CTD‐ILD. CTD‐ILD patients are a heterogenous group with a wide spectrum of autoimmune conditions including systemic sclerosis (SSc), rheumatoid arthritis (RA), inflammatory myopathies and primary Sjogren’s syndrome. They remain a significant treatment challenge because of their differing (and sometimes multiple) patterns of fibrotic disease, variable rates of progression and limited available clinical data on best time to intervene. Many CTD‐ILD patients can remain stable for years whilst not on therapy but conversely respiratory failure remains a leading cause of death most notably in the SSc cohort and second in RA.^64^ The new IPAF group is further complicated as there is no evidence to guide treatment in this cohort. Until recently, they have been defined for research purposes rather than recognised as a separate clinical entity, indicating there is a need for further evaluation of this group.

Significant variability regarding which immune‐modulating agent, dose and treatment duration exists across the board because of lack of available consensus guidelines. To date, there has been no evidence of superiority amongst the use of azathioprine, mycophenolate, cyclophosphamide or cyclosporine in CTD‐ILD. Choosing immunosuppressant therapy is therefore still merited on an individual basis. In clinical practice, most are used as additional therapy to glucocorticoids to enable the lowest clinically effective dose of steroid (ideally < 10 mg daily).^65^ Whilst some therapies may be chosen based on their superior effects on extrapulmonary systems (e.g. mycophenolate in sclerodactly), the best treatment is still not clear in many of these clinical entities. Therefore, it is imperative, both for individual patients and for ongoing trials that the type of disease is defined as well as possible.

By far, the most studied CTD‐ILD population is the SSc group with data extrapolated to guide treatment of other CTD‐ILD. In the Scleroderma Lung Study (SLS 1), 158 patients with SSc‐ILD were randomised to receive cyclophosphamide versus placebo.^66^ The mean decline from baseline in FVC % predicted was 1.0% in the cyclophosphamide group versus 2.6% in the placebo group (*P* < 0.05). Concerns regarding side effects and tolerability prompted the SLS II trial to directly compare cyclophosphamide (for 12 months followed by 12 months placebo) with mycophenolate mofetil (for 24 months), with both drugs demonstrating similar improvements in FVC but significantly fewer side effects and lower dropout rates in the mycophenolate group despite the extended duration of use.^67^ Rituximab, an anti‐CD20 monoclonal antibody, has been used predominantly as a rescue therapy in refractory CTD‐ILD and has shown promise in several case series in both SSc‐ILD‐associated and anti‐synthetase‐associated ILD.^68^, ^69^ The RECITAL (Rituximab versus Cyclophosphamide) trial is a multicentre, randomised, double‐blind controlled trial currently recruiting in the UK to assess the efficacy of rituximab as first‐line treatment in CTD‐ILD.^70^

Announced earlier this year were the results of the much anticipated SENSCIS trial, a randomised placebo‐controlled trial, which assessed nintedanib versus placebo in SSc‐ILD over 52 weeks.^71^ Whilst a modest reduced rate of annual FVC decline (−52 mL year^1^ in nintedanib versus −93.3 mL year^1^ in placebo, *P* = 0.04) was reported in the nintedanib group, there was a high proportion of reported adverse events of which diarrhoea was most common (75.7% in nintedanib group and 31% in placebo respectively). Interestingly, almost 50% of patients were receiving mycophenolate prior to enrolment, challenging discussion as to whether the combination of anti‐fibrotic and immunosuppressant therapy is of additional benefit in this cohort.

## Future direction

With ever evolving understanding of the innate fibrotic processes across the various ILD subtypes, changes in treatment will inevitably follow. Such an approach is the focus of a current placebo‐controlled trial entitled Progressive Fibrosing‐ILD (INBUILD study), comparing nintedanib with placebo. This trial excludes patients with IPF but will include patients with UIP‐like pattern (such as in CTD) or non‐UIP pattern (which include NSIP, HP and sarcoid).^53^ In tandem, there is a double‐blind, randomised, placebo‐controlled phase II trial assessing pirfenidone in patients with unclassifiable disease, importantly inclusive of IPAF, and will allow the concomitant use of mycophenolate (NCT03099187).

New agents targeting the various stages in the pathogenesis (Figure 3) of disease are under study in IPF and include calpain inhibitors, which are calcium‐dependent cysteine proteases that influence cell signalling; metformin, which activates AMP‐activated protein kinase facilitating deactivation and apoptosis of myofibroblasts potentially reversing established fibrosis; and GLPG1690, a novel inhibitor of autotaxin, an enzyme involved in lysophosphatidic acid (LPA) production. A phase 2a randomised placebo‐controlled trial with GLPG1690 showed favorable stability of FVC (although not sufficiently powered) compared to placebo with good safety profile.^72^ BMS‐986020, an LPA receptor antagonist, has demonstrated a slower rate of FVC decline in a recent Phase 2 double‐blinded randomised control trial.^73^ Another novel drug PBI‐4050 targeting transforming growth factor 1 β (TGF‐1β), connective tissue growth factor (CTGF) and cytokines IL‐23p19 and IL‐6 has successfully navigated a Phase 2 trial of safety and efficacy with no concerns reported.^74^ This trial allowed concurrent use of either pirfenidone or nintedanib with stable FVC (over a 12‐week period) reported in both PBI‐4050 alone and PBI‐4050/nintedanib but reduced in PBI‐4050/pirfenidone cohort.

Several trials are currently underway exploring combination therapy with anti‐fibrotics and immunosuppression in progressive fibrotic disease of any aetiology, with particular focus on the CTD‐ILD cohort. SLS III is currently recruiting SSc‐ILD patients to trial combination therapy with mycophenolate and pirfenidone. Other forthcoming trials include TRAIL1: Phase ll Study of pirfenidone in Patients with RA‐ILD (NCT02808871) and, as discussed, RECITAL: Rituximab versus Cyclophosphamide in Connective Tissue Disease‐ILD (NCT01862926). A favorable outcome in any or all of these trials has significant potential to change practice relating to anti‐fibrotic use outside the IPF cohort.

## Summary

Comprehensive clinical, morphological and serological assessment by a multidisciplinary expert team in a combined meeting is now the standard of care expected for all patients with ILD requiring monitoring or treatment. The team should be up‐to‐date with evolving criteria for diagnosis and new investigations, but ultimately patients are categorised according to their clinical phenotype, using ratings of strength of evidence to support a diagnosis. These can guide the need for further testing and increase confidence of diagnosis. Genotyping demonstrates great heterogeneity in clinical patterns and at this stage is not helpful other than the finding of short telomeres which indicate greater complications post‐transplantation.

Broadly, ILD treatment can be divided into four domains: (1) observation and monitoring; (2) disease‐modifying therapies such as anti‐fibrotics, immunosuppression and lung transplantation; (3) management of co‐morbidity including gastro‐oesophageal reflux, cough and mental health; and (4) non‐pharmacological strategies such as pulmonary rehab, oxygen and education. Palliative care discussion should be encouraged at any stage in the disease focusing on both symptom control and, when needed, end‐of‐life care. The anti‐fibrotic era has been exciting, proving that rate of disease progression can be slowed and exacerbations can be reduced, although their use is currently limited to the IPF cohort.

The question remains as to whether we should be characterising disease, and therefore choosing treatment, based on the clinical phenotype or the course of the disease (progressive vs non‐progressive) or the stage of the disease (inflammation in early stage in CTD‐ILD, fibrosis with architectural distortion or honeycombing in the late phases). The question of targeting process or disease pattern instead of specific disease entities is already being challenged^52^ and being explored in the progressive disease phenotype studies.^53^ With the additional prospect of new drug compounds such as calpain inhibitors or autotaxin inhibitors, an exciting paradigm shift in ILD management is awaited.

## Conflict of interest

IM has received funding to attend an educational meeting from Boerigher Ingelheim, the manufacturers of nintenadib. AMT and FL declare no conflicts of interest.
